# Human dose response relation for airborne exposure to *Coxiella burnetii*

**DOI:** 10.1186/1471-2334-13-488

**Published:** 2013-10-21

**Authors:** Russell John Brooke, Mirjam EE Kretzschmar, Nico T Mutters, Peter F Teunis

**Affiliations:** 1 Julius Center for Health Sciences and Primary Care, University Medical Center Utrecht, Utrecht, The Netherlands; 2 Centre for Infectious Disease Control, RIVM, Bilthoven, The Netherlands; 3 Department of Infectious Diseases, Medical Microbiology and Hygiene, Heidelberg University Hospital, Heidelberg, Germany; 4 Hubert Department of Global Health, Rollins School of Public Health, Emory University, Atlanta, GA, USA

**Keywords:** Q fever, Dose response, Challenge study, Risk analysis, Beta-poisson

## Abstract

**Background:**

The recent outbreak of Q fever in the Netherlands between 2007 and 2009 is the largest recorded Q fever outbreak. Exposure to Coxiella burnetii may cause Q fever but the size of the population exposed during the outbreak remained uncertain as little is known of the infectivity of this pathogen. The quantification of the infectiousness and the corresponding response is necessary for assessing the risk to the population.

**Methods:**

A human challenge study was published in the 1950s but this study quantified the dose of C. burnetii in relative units. Data from a concurrent guinea pig challenge study were combined with a recent study in which guinea pigs were challenged with a similar aerosol route to quantify human exposure. Concentration estimates for C. burnetii are made jointly with estimates of the dose response parameters in a hierarchical Bayesian framework.

**Results:**

The dose for 50% infection (InfD50%) in human subjects is 1.18 bacteria (95% credible interval (CI) 0.76-40.2). The dose for 50% illness (IllD50) in challenged humans is 5.58 (95%CI 0.89-89.0) bacteria. The probability of a single viable C. burnetii causing infection in humans is 0.44 (95%CI 0.044-0.59) and for illness 0.12 (95%CI 0.0006-0.55).

**Conclusions:**

To our knowledge this is the first human dose–response model for C. burnetii. The estimated dose response relation demonstrates high infectivity in humans. In many published papers the proportion of infected individuals developing illness is reported to be 40%. Our model shows that the proportion of symptomatic infections may vary with the exposure dose. This implies that presence of these bacteria in the environment, even in small numbers, poses a serious health risk to the population.

## Background

The Netherlands recently underwent the largest Q fever outbreak on record with more than 3500 notified cases between 2007 and 2009 [1] and possibly more than 40,000 infections [2] with a substantial disease burden (Brooke et al., manuscript under consideration). The outbreak was associated with an increase in intensive goat farming and attributed to the release of bacteria during multiple abortion storms in the region [3,4]. Mass culling and subsequent vaccination of goats halted the outbreak but whether the effects of these measures are permanent is still unknown as background incidence remains above pre-outbreak levels [4].

In order to better understand the relation between human exposure to *Coxiella burnetii* released into the environment near infected goat farms, and the risk of subsequent health effects, efforts are ongoing to quantify the risk of becoming infected or developing acute symptoms given exposure to *C. burnetti*. A dose response model describes the functional relationship between the dose (the magnitude of exposure) and the probability of infection and/or acute illness, allowing translation of exposure estimates into risk estimates. Although dose response relations have been published for several microbial pathogens based upon human challenge studies, such information is normally not available for pathogens that can cause severe illness, such as *C. burnetii*.

During the cold war in the 1950s and 1960s, the United States military performed human challenge studies for many biological agents that were deemed a plausible biological weapon [5]. One of these studies [6] established a dose response relationship in humans for *C. burnetii*. However, in this study the challenge dose was not expressed in numbers of bacteria but as serial dilutions of a *C. burnetii* suspension in egg slurry. Concurrent to the human challenge experiment a guinea pig challenge experiment was performed with the same methodology and materials. More recently, Russell-Lodrigue et al. performed a similar experiment on guinea pigs with a quantified dose of *C. burnetii* expressed in numbers of bacteria [7]. In this paper we combine these studies to estimate the infectious dose for the human challenge study in numbers of bacteria and establish a human dose response model for *C. burnetii*.

## Methods

### Data

The original challenge studies by Tigertt et al. [6] used egg slurry infected with the AD strain of *C. burnetii*, diluted with water, aerosolized in an aerosol chamber of known size to study the exposure-response relationship. Infection was defined as seroconversion in a patient by standard complement fixation 10 to 20 days after illness. In addition, clinical symptoms were recorded for all patients. Clinical symptoms of a typical infection included the onset of persistent temperature above 38°C (100°F), lassitude, loss of appetite, headache, mild photophobia, bradycardia, and occasionally a palpable spleen. Chest radiography (x-ray) showed evidence of pneumonia in roughly half of patients developing clinical illness though no cough or rhonchi were observed and the size of lesions were not related to dose. The data used is taken from the original articles (Table 1). The more recent challenge study in guinea pigs [7] used bacterial challenge doses quantified by commercially available bacterial viability kit (Live/Dead BacLight bacterial viability kit Molecular Probes, Eugene, OR) administered through a specialized apparatus to deliver aerosol uniformly within an exposure chamber [7,8].

**Table 1:** Datasets from original articles used to estimate dose response relationships

**Tigertt guinea pig**				**Tigertt human**				**Russell-Lodrigue guinea pig**			
**Egg slurry**	**E**	**I**	**S**	**Egg slurry**	**E**	**I**	**S**	**Bacteria**	**E**	**I**	**S**
10^-6^	29	0	-	10^-6^	2	0	0	2 x 10^1^	3	3	2
10^-5^	26	1	-	10^-5^	5	4	2	2 x 10^2^	3	3	3
10^-4^	24	4	-	10^-4.5^	3	3	3	2 x 10^3^	3	3	3
10^-3^	27	22	-	10^-4^	8	7	7	2 x 10^4^	3	3	3
10^-2^	28	28	-	10^-3^	5	4	4	2 x 10^5^	3	3	3
10^-1^	24	24	-	10^-2^	4	4	4	2 x 10^6^	3	1^*^	3
				10^-1^	2	2	2				

*Two of the guinea pigs died before the end of the study when seroconversion was determined.

Egg slurry - dilutions of 1 milliliter of infected egg slurry with milliliters of sterile water.

Bacteria - a Poisson process exposure number of bacteria.

E - number of exposed per exposure group.

I - number of infected individuals per exposure group.

S - number of symptomatic per exposure group.

When estimating the aerosol dose in the Tigertt study the different respiratory rates of guinea pigs and humans need to be taken into account. The estimated dose is calculated using the concentration of bacteria in the aerosol and the volume of aerosol inhaled during the one minute period of exposure. Respiratory rates of infectious aerosol were assumed to be a log-normal distributed; 5–10 *l* (geometric mean (GM) 7.1 *l*) for humans [9] and 0.1-0.35 *l* (GM 0.19 *l*) for guinea pigs [10].

The Russell-Lodrigue et al. study uses particle counts to determine the number of particles in the stock solution after purifying the Nine Mile (RSA 493) strain. Serum samples were tested by ELISA and clinical signs recorded under direct observation during the study period. Clinical signs, such as increased respiratory rates and sounds were recorded as well as fever and histopathological changes to relevant organs.

### Model

The dose response relation used to predict infection is a hit theory model that assumes Poisson exposure and a variable (beta distributed) probability of a pathogen surviving all host barriers to infection [11]. Poisson distributed exposure implies that the inhalation of a volume *V* from a suspension of concentration *c* results in exposure to a discrete random number of bacteria that is Poisson distributed with an expected value of *cV*. Heterogeneity in the host-pathogen interaction is taken into account by assuming that the variable probability of any individual infectious particle succeeding in surviving all host barriers and causing infection, is beta distributed [11]. The resulting dose response relation is

$$P_{\inf}\left(\mathrm{cV}|\alpha ,\beta \right)=1{-}_{1}F_{1}\left(\alpha ,\alpha +\beta ;\mathrm{cV}\right)$$

where _1_*F*_1_() is a confluent hypergeometric function [12] and (*α,β*) are the parameters of the beta distribution describing heterogeneity. Infection may not always be symptomatic: a fraction of those infected may develop symptoms of acute illness and that fraction may again depend on the dose [13]. To account for a dose dependent probability of symptoms we have used the conditional illness model proposed by Teunis et al. [13].

$$P_{\mathrm{ill}|\inf}\left(\mathrm{cV}|\eta ,\rho \right)=1-{\left(1+\mathrm{\eta cV}\right)}^{-\rho }$$

With parameters (*η,ρ*) respectively describing the scale and shape of the conditional illness model [13]. The dose response data consist of groups of subjects (*N*) exposed to different doses resulting in numbers infected (*K*) or symptomatic (*S*), leading to a binomial likelihood

$$\ell \left(\alpha ,\beta \right)=P_{\inf}^{K}{\left(1-P_{\inf}\right)}^{N-K}$$

$$\ell \left(\eta ,\rho \right)=P_{\mathrm{ill}}^{S}{\left(1-P_{\mathrm{ill}}\right)}^{K-S}$$

where the probability of infection (*P*_inf_) depends on the dose. The parameters for infectivity (*α, β*) were transformed to improve convergence (see Teunis et al. [14]) and for illness (*η, ρ*) parameters were log normal distributions, Additional file 1: code S1.

A useful measure for infectivity is the 50% infectious dose (InfD50). This is the dose where 50% of the exposed are expected to become infected based upon the dose response relationship. Also, the 50% illness dose (IllD50) where an estimated 50% of the exposed subjects develop acute Q fever symptoms is useful. In addition, the ratio of asymptomatic cases to a single symptomatic case, which is used to estimate the total number of infected from notified cases, can indicate differences in attack rates within populations of different exposures. Using outbreak data one could calculate backward from attack rates within a population to make estimates about exposure.

Simultaneous estimation of parameters for the dose response relations and the dilution factor relating the doses of Tigertt et al. and Russell-Lodrigue et al. is most conveniently done in a Bayesian framework by obtaining the joint posterior probability of parameters from the combined data sets and optimizing by means of Markov chain Monte Carlo (MCMC). A graphical representation (DAG: directed acyclic graph) of the model structure and dependencies, see Additional file 1.

The model was implemented in JAGS (v3.2.0) and run using rjags (v3-5) in R (v2.15.1).

## Results

**Figure 1:**
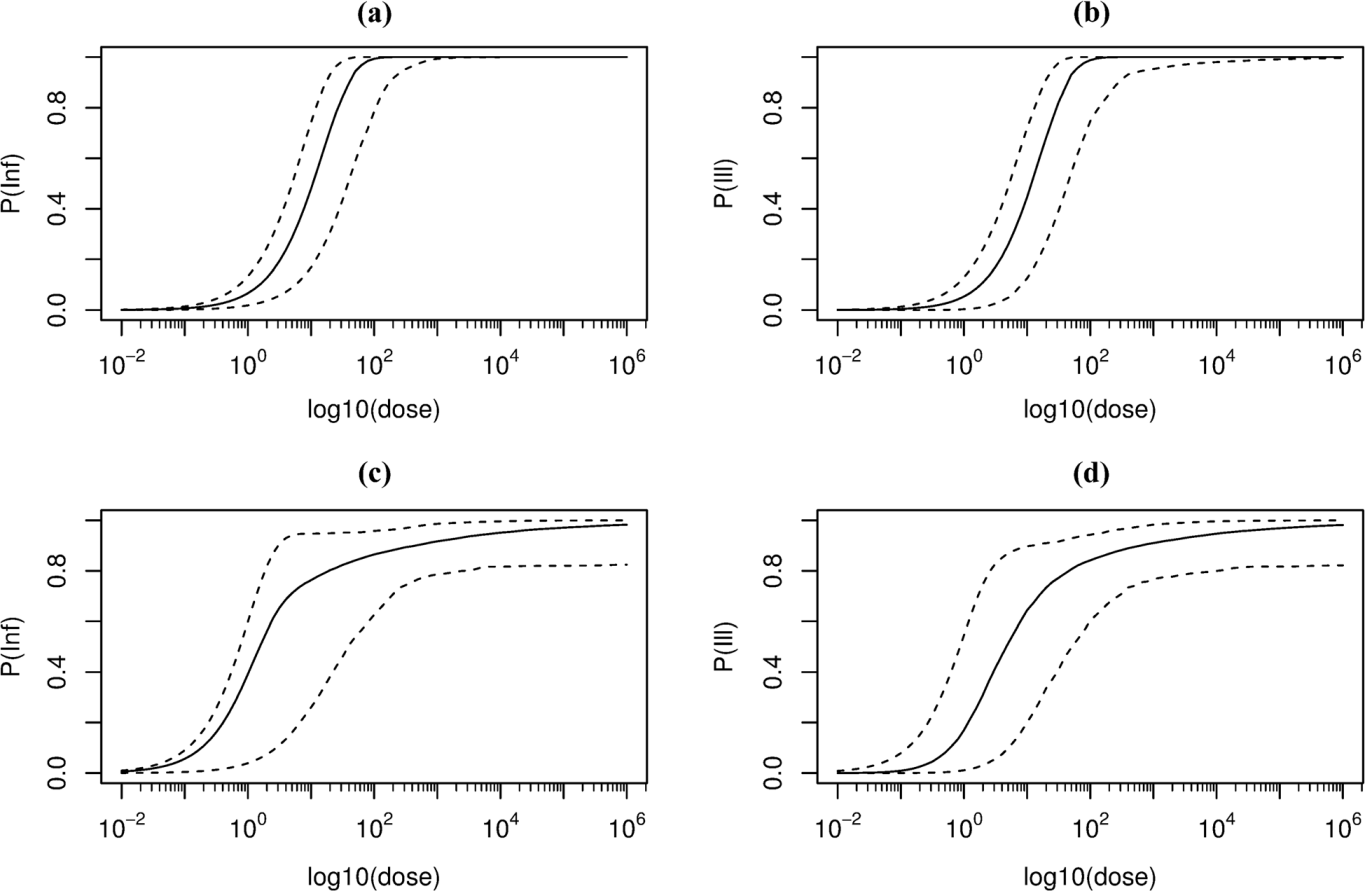
**Dose response relations for C. burnetii.** Median probability and 95% range as a function of dose: infection in guinea pigs **(a)**, symptoms of acute illness in guinea pigs **(b)**, and infection **(c)** and acute illness symptoms **(d)** in humans.

**Figure 2:**
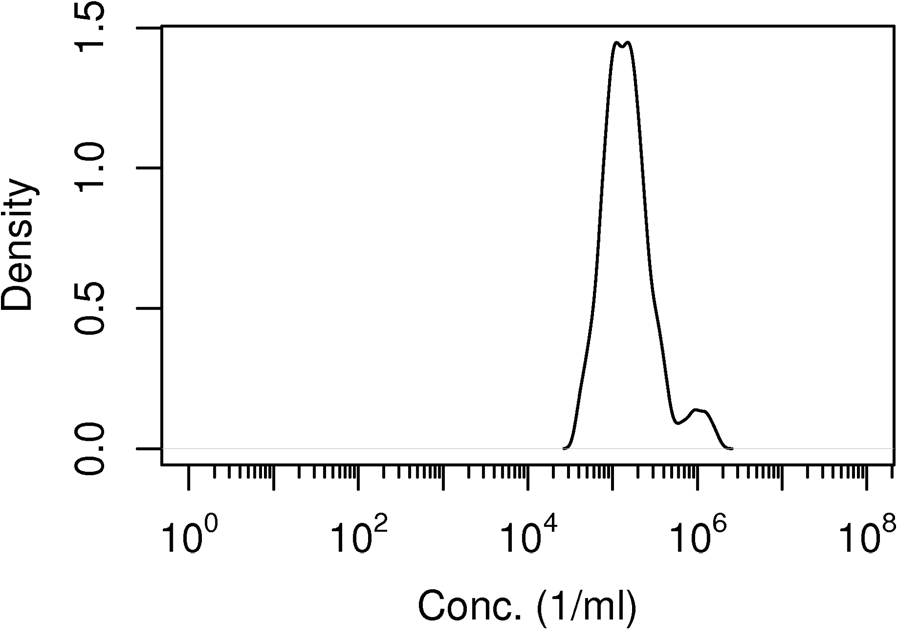
**Estimated concentration of C. burnetii in the inoculum used in the human challenge study.** Estimated posterior probability density.

The model was run for 5×10^7^ iterations with a burnin of 1×10^6^ and thinning of 5×10^4^ iterations respectively; convergence was checked (Gelman test, CODA [15]). Parameter estimates for the model are briefly summarized (Table 2) and predicted outcomes by the model are reported (Table 3). Dose response relations for guinea pigs, based on joint data from the Tigertt and Russell-Lodrigue studies, were steep, indicating little heterogeneity in infectivity (Figure 1a and b). Both guinea pig and human dose response relations show considerable risk of infection and illness at low doses (Figure 1c and d); at high doses a small fraction of humans appear to be protected against infection, as indicated by the slow increase of the infection (and illness) probabilities with doses above a count of 1000 live particles, which is similar to colony forming units for other pathogens. The median probability of infection from a single bacterium in humans is 0.44 (95%CI 0.044-0.59) and the median probability of illness 0.12 (95%CI 0.0006-0.55) (Table 4). In guinea pigs the median probability of infection is 0.06 (95%CI 0.016-0.14) and the median probability of illness 0.04 (95%CI 0.0003-0.13). The median dilution factor for the Tigertt study challenge doses, representing the concentration difference between the two units of the study, is 142000 milliliters water to one milliliter infected egg slurry (95% CI 47874–1075165) (Figure 2); a single infectious particle in the Russell-Lodrigue study is estimated to be equivalent to the same as 1 milliliter infected egg slurry homogenously mixed with 142 liters of water.

**Table 2:** Parameter estimates for infection and illness dose response of posterior MC estimates

	***α***		***β***	
	**Median**	**95% range**	**Median**	**95% range**
Guinea pigs	14.25	1.23-8.78x10^3^	203.23	11.94-1.10x10^5^
Humans	0.23	6.82x10^-5^-0.71	0.18	1.05x10^-5^-10.52
	***η***		***ρ***	
	**Median**	**95% range**	**Median**	**95% range**
Guinea pigs	1.34	0.0016-1.82x10^4^	24.51	0.31-1.00x10^5^
Humans	0.88	8.32x10^-3^-2.9x10^4^	6.88	0.23-6.79x10^3^

α - shape parameter of the infection beta-Poisson function.

β - scale parameter of the infection beta-Poisson function.

η - shape parameter of the illness beta-Poisson function.

ρ - scale parameter of the illness beta-Poisson function.

**Table 3:** Predicted number of outcomes for the data by exposure group (E), predicted infected (IP), and predicted symptomatic (SP)

**Tigertt guinea pig**				**Tigertt human**				**Russell-Lodrigue guinea pig**			
**Egg slurry**	**E**	**IP**	**SP**	**Egg slurry**	**E**	**IP**	**SP**	**Bacteria**	**E**	**IP**	**SP**
10^-6^	29	0.28 (0.12-0.73)	0.2 (0.001-0.69)	10^-6^	2	0.13 (0.03-0.42)	0.04 (0–0.27)	2 x 10^1^	3	2.14 (0.92-2.79)	2.05 (0.80-2.73)
10^-5^	26	2.37 (1.02-5.71)	2.13 (0.06-5.59)	10^-5^	5	2.09 (0.61-4.02)	1.12 (0.16-3.39)	2 x 10^2^	3	2.99 (2.75-3.00)	2.99 (2.56-3.00)
10^-4^	24	14.4 (7.85-20.65)	13.85 (3.35-20.61)	10^-4.5^	3	2.03 (0.87-2.77)	1.43 (0.44-2.57)	2 x 10^3^	3	3 (2.99-3.00)	3 (2.89-3.00)
10^-3^	27	26.96 (25.56-27)	26.91 (22.63-27)	10^-4^	8	6.26 (4.09-7.57)	5.50 (2.97-7.12)	2 x 10^4^	3	3.00 (3.00-3.00)	3.00 (2.95-3.00)
10^-2^	28	28 (27.92-28)	28 (26.83-28)	10^-3^	5	4.39 (3.80-4.80)	4.30 (3.67-4.74)	2 x 10^5^	3	3.00 (3.00-3.00)	3.00 (2.98-3.00)
10^-1^	24	24 (24–24)	24 (23.50-24)	10^-2^	4	3.71 (3.23-3.95)	3.69 (3.13-3.94)	2 x 10^6^	3	3.00 (3.00-3.00)	3.00 (2.99-3.00)
				10^-1^	2	1.91 (1.63-1.99)	1.91 (1.61-1.99)				

Egg slurry - dilutions of 1 milliliter of infected egg slurry with milliliters of sterile water.

Bacteria - a Poisson process exposure number of bacteria.

E - number of exposed per exposure group.

IP - model predicted number of infected individuals per exposure group.

SP - model predicted number of symptomatic per exposure group.

**Table 4:** Estimated Poisson dose for 50% probability of infection and for 50% probability of acute illness

	**InfD50**		**IllD50**	
	**Median**	**95% range**	**Median**	**95% range**
Guinea pigs	10.69	5.04-38.42	11.79	5.26-44.13
Humans	1.54	0.75-38.69	4.68	0.87-51.00

InfD50 - the Poisson dose for 50% probability of infection.

IllD50 - the Poisson dose for 50% probability of illness.

**Figure 3:**
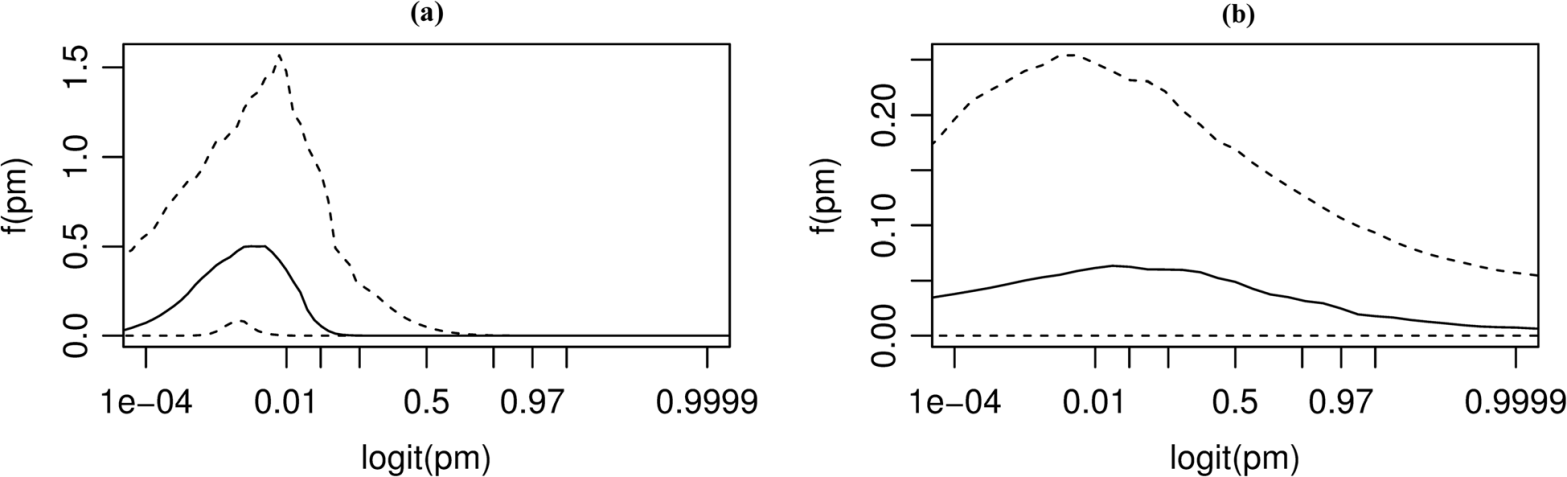
**Distribution of the probability of infection per single infectious particle.** Characterization of susceptibility to infection with C. burnetii in guinea pigs **(a)** and in human volunteers **(b)**. Median and 95% range of posterior probability density.

The variability in the infection risk from a single particle, as described by its (beta) distribution, is shown in Figure 3.

**Figure 4:**
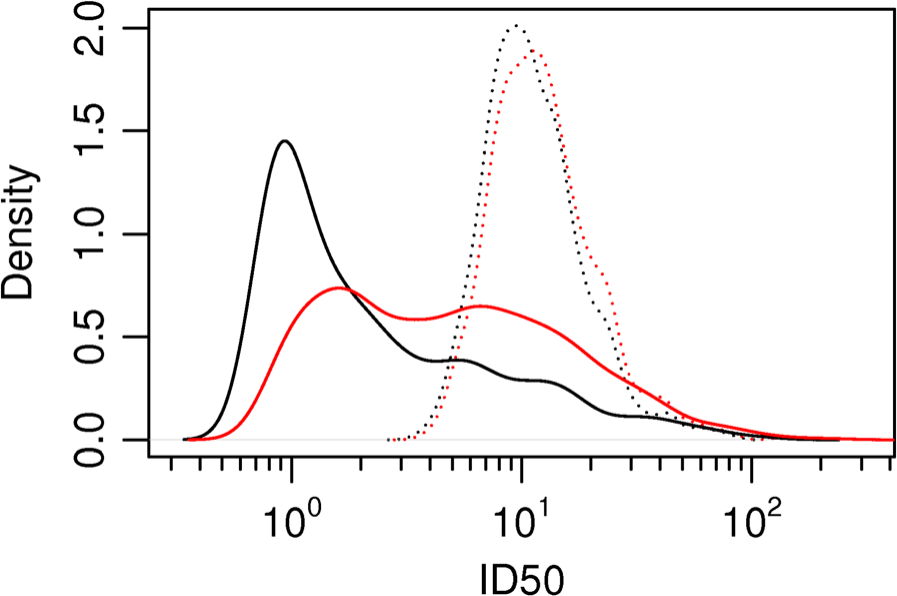
**Estimated dose where probability of infection is 50% and probability of acute respiratory symptoms is 50%.** Guinea pigs infection hatched black curve, human infection solid black curve, guinea pigs illness hatched red curve, humans illness solid red curve.

Plotting the distribution of the InfD50 and IllD50 estimates from the data provides additional information about the uncertainty of dose response relationship. The guinea pigs have a less heterogeneous response than humans, as shown in the narrow distributions of InfD50 and IllD50 (Figure 4 - hatched lines). Estimates of human data InfD50 and IllD50 show wide ranges, indicating that risk of infection and illness may be more variable (Figure 4 – solid lines).

**Figure 5:**
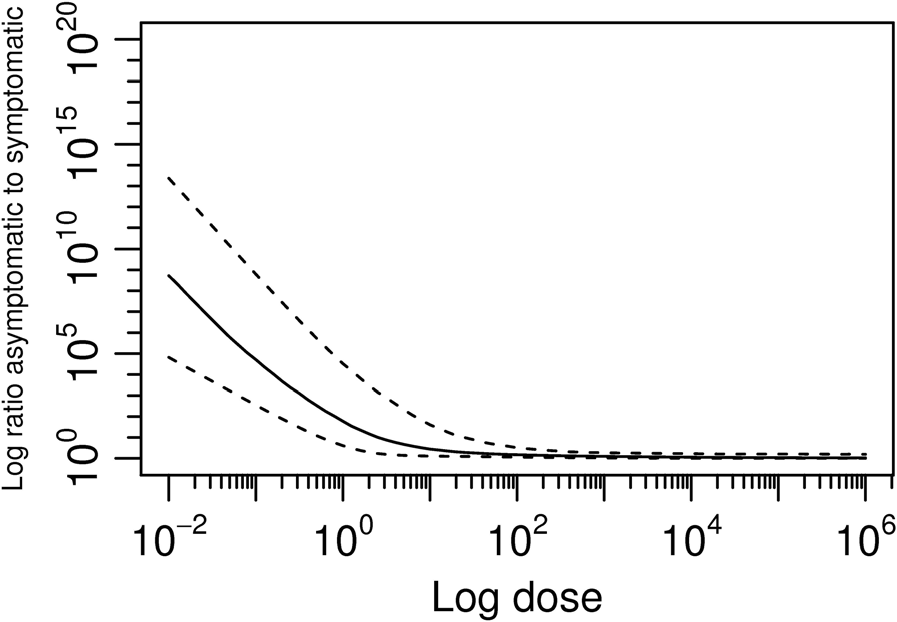
Estimated ratio infected cases to symptomatic cases.

The ratio of the human infected to ill ratio indicating number of asymptomatic infections to symptomatic infections decreases as the probability of illness increases (Figure 5). As the dose increases the probability of infection increases as does the probability of developing illness.

An overview of the parameters, their prior distributions, the model parameter estimations and model code provides information about the model structure and the Bayesian framework (Additional file 1).

## Discussion and conclusions

To our knowledge this is the first human dose–response model for *C. burnetii* that quantifies the exposure-outcome relationship. Per single bacterium the median probability of infection in humans is 0.44 (95%CI 0.044-0.59) and for illness 0.12 (95%CI 0.0006-0.55), variability in the credible intervals is observed in the probability density of infection of a single bacteria, Figure 3. Humans appear more susceptible than guinea pigs to infection with *C. burnetii* however with greater uncertainty. The cause may be the specially bred Hartley guinea pigs, used both in Tigertt’s and Russell-Lodrigue’s research, that are bred to reduce genetic heterogeneity and therefore similar in their response. In humans the InfD50 and the IllD50 are more disperse than in guinea pigs indicating that there are other (unknown) host factors that are affecting the dose response relation.

In many published papers the proportion of infected individuals developing illness is reported to be 40% [16]. Our model shows that the proportion of symptomatic infections may vary with the exposure dose. Data from the Dutch outbreaks report different proportions of asymptomatic infections, which may be due to different exposure levels [2,17]. In outbreaks with a well defined point source [3,18] the distance from the outbreak source is negatively correlated with the symptomatic (attack) rate. This may be partly due to the dispersion of the aerosol over a larger area leading to lower exposure levels at increasing distance from the source. At lower doses the risk of infection decreases but the fraction of those infected who become ill also decreases and thus the number of cases are expected to drop even more rapidly with distance from the source. Failing to take into account the increase in proportion of asymptomatic cases as the exposure decreases may lead to underestimation of the total number of individuals infected in an outbreak.

At a low average dose of 0.1, as may be associated with an exposure outside of a clinical setting, the median risk of becoming ill is 0.006 (95%CI 0.00003-0.08) while the median probability of infection is 0.06 (95%CI 0.005-0.09). As a consequence, when spatial and temporal clustering of cases is found, exposure to high numbers of bacteria may be presumed: such recognizable clusters of cases indicate severe exposure. However, when low numbers of the pathogen are released, exposed subjects may still be infected (due to their high susceptibility) but far fewer of those infected develop acute symptoms. Therefore, such low-level (endemic) infections tend to cause isolated cases: the absence of clustering makes detection of the source difficult. The demographics of an exposed population are also important as men appear to be symptomatic more frequently than women [19] while infection appears gender independent [17]. Also, symptomatic rates in age groups vary, increasing and peaking around 60 years of age [19]. Whether these differences arise from exposure differences or physiological differences would need further investigation to validate the use of this dose response relationship for exposure quantification. However, once this has been incorporated into the dose response relationship one could estimate exposure from infection attack rates of infection and illness. One of the first outbreaks in the Netherlands with a documented point source outbreak with a high attack rate (17.59% of population acutely infected) may be used to calculate crude estimates of exposure [18]. According to this estimate, exposure is low, median 0.30 bacteria (95%CI 0.21-5.67). Note that this means that on average one in three individuals was exposed to a single bacterium.

A recent review of animal models for infectivity of Q fever concluded that to estimate human infectivity pathogen aerosol challenge data was required [20]. Animal studies are not appropriate for studying human infectivity as the animal hosts used are frequently immunodeficient and the infection pathway is frequently not representative of natural infection. One study used three different mouse strains injected intraperitoneally and report a LD50 (50% lethal dose) of 2302 organisms [21]. The authors suggest that severe combined immunodeficient (SCID) mice would be a good model for chronic Q fever as immunodeficiency is a risk factor for chronic Q fever in humans. Our results illustrate that immune competent humans inoculated “naturally”, via aerosol, are much more susceptible to *C. burnetii* infection than these immune deficient mice inoculated intraperitoneally (presumably bypassing respiratory barriers to infection). Use of animals as surrogate hosts for dose response assessment in humans can be misleading, as there is little quantitative knowledge of how to translate susceptibility across host species and/or inoculation routes. In a study on the dose response relation for *E. coli* O157:H7 comparing an animal challenge model to human response data, a strikingly similar pattern was found: human susceptibility was much higher than the estimate from the animal model [22]. The paper of Tigertt et al. [6] can be considered a model study for how to perform and interpret animal dose response data by reporting a joint study in humans and animals, using the same inoculum. See also DuMouchel and Harris [23] for a proposed approach to analyze such combined data.

The guinea pig challenge experiments of Tigertt et al. and Russell-Lodrigue et al. have many similarities but the two studies do use different strains of *C. burnetii*: AD strain of *C. burnetii* (isolated from milk in California) and the Nine Mile phase 1 (RSA 493) respectively. Our titration of the suspension used by Tigertt et al. is based on the assumption that the two strains are equally infectious in guinea pigs. Other studies have attempted to identify if there are differences in infectivity between strains but with doses of many orders of magnitude higher, immune deficient animal hosts, lipoprotein antigens instead of bacteria, and/or mortality instead of infection as an outcome [21,24,25]. There are no studies of strain infectivity in guinea pigs or with aerosol challenge models. Another assumption is that the serological tests used, complement fixation (CF) in the Tigertt et al. study and enzyme-linked immunosorbent assay (ELISA) in the Russell-Lodrigue et al. study, are similar in their sensitivity and specificity. A study comparing the two tests show they have comparable sensitivity (100%/100% vs 100%/67% for phase II and phase I respectively) and specificity (100%/100% vs 95%/95%) for IgG [26]. There is no information about clustering or aggregation in the studies but the beta distribution provides information regarding the variation in infectivity of individual infectious units, Figure 3, part of which may reflect variation in the numbers of infectious bacteria per particle. The visible effect of such clumping is that it adds heterogeneity and would make the dose response relationship less steep. Unpublished observations of natural settings are usually close to detection limits of real-time polymerase chain reaction (qPCR), which suggests that when present the bacteria are more likely single units than clumps of multiple bacteria. However, sensitivity and specificity of the used assays may affect these results and their interpretations.

A methodological issue with the study, compared to a clinical trial, is the low number of cases in the different challenge dose groups. However, human challenge studies are rarely very large and despite the small group numbers the dose response relation is very well defined. Additions to the framework, including additional data sources from natural experiments, may allow further refinement of the dose response relationship though these have their own issues regarding exposure quantification and exposure time. A second methodological issue is the assumption that none of the challenged subjects have been previously infected. The United States’s national Q fever seroprevalence was recently estimated at 3.1% [27] and though subjects may seroconvert, antibody levels measured before being challenged may have been below detection level. If an immune subject were included in the experiment, an expected outcome might be to observe a seroconversion but not develop illness. In the study with human subjects, two cases were observed to seroconvert without illness. To test what the effects of missing such a case would be, a sensitivity analysis was performed by increasing the positive responses in the challenge groups (infection and illness) by one (scenario 1) or by two individuals (scenario 2). Resulting estimates were similar, unless the scenario led to groups with 100% infection. The model was also run without any data in the model and the posterior distributions of the parameters did not change significantly (Additional file 1: Table S1).

The experimental study of the illness dose response includes mild cases as well as severe cases, which are more likely to be identified and notified. In the Netherlands, a watchful waiting period of two weeks, the average duration of acute Q fever, is frequently employed for febrile illnesses. In the Tigertt study the duration of symptoms and the length of the incubation period of challenged subjects were dose dependent. This will affect the number of notified cases as individuals with a low dose and mild illness will be resolved within the watchful waiting period. The dose response relationship for humans indicates different infection rates for different doses; high infectivity is observed at low bacterial levels while higher doses increase the probability of infection illness. The more than 3500 notified cases may only represent a small proportion of the number infected in the Netherlands and estimates of 40,000 infected may not be exaggerated.

## Competing interests

The authors declare that they have no competing interests.

## Authors’ contributions

RB performed the literature review, data gathering, statistical analysis and the writing of the manuscript. MK performed the literature, statistical analysis and the drafting of the manuscript. NM performed the data gathering, clinical and research analysis and drafting of the manuscript. PT performed the literature review, statistical analysis, and drafting of the manuscript. All authors read and approved the final manuscript.

## Pre-publication history

The pre-publication history for this paper can be accessed here:

http://www.biomedcentral.com/1471-2334/13/488/prepub

## Supplementary Material

Additional file 1: Figure S1 Simplified directed acyclic graph of the model without priors. **Table S1**. Prior distributions (mean, sd) in the Bayesian framework, posterior distributions and posterior distributions of the model without data. Code S1 JAGS model representing the Bayesian framework. Click here for file
