# Supplementary material for: Human dose response relation for airborne exposure to Coxiella burnetii
Source: BMC Infect Dis. 2013 Oct 21;13:488. doi: 10.1186/1471-2334-13-488 (PMC3827992; doi:10.1186/1471-2334-13-488)
Supplement: Additional file 1: Figure S1 — Simplified directed acyclic graph of the model without priors. Table S1. Prior distributions (mean, sd) in the Bayesian framework, posterior distributions and posterior distributions of the model without data. Code S1 JAGS model representing the Bayesian framework. [file 1471-2334-13-488-S1.docx]

**Additional files:**

***Figure SM1 Simplified graph of model without priors.***


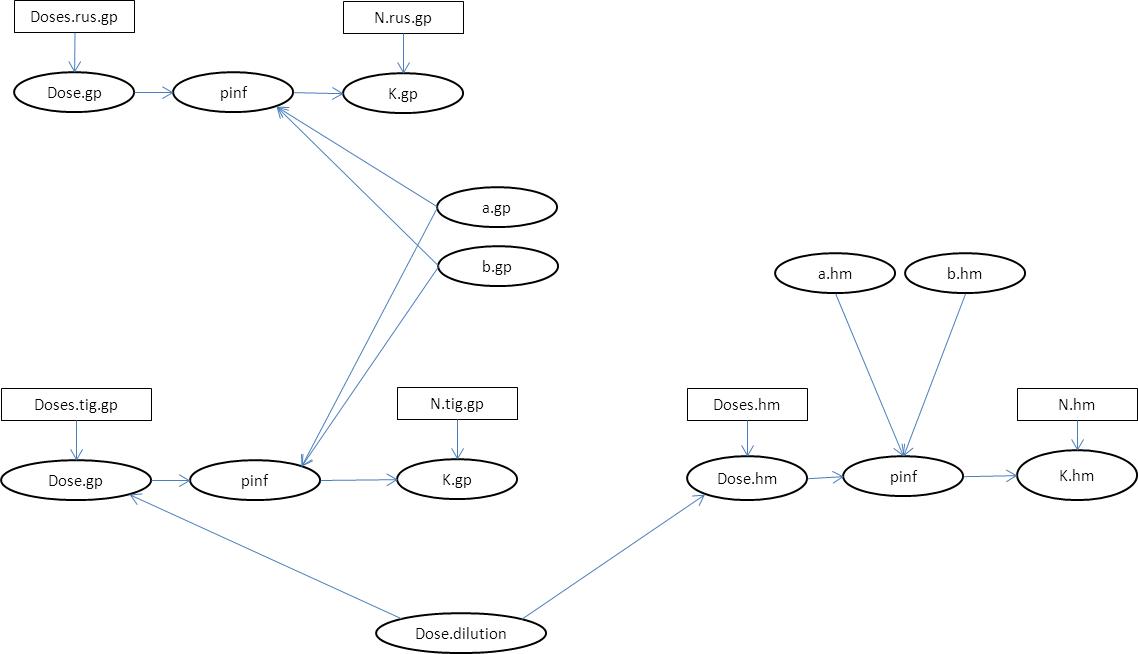


| Legend | | | |
| --- | --- | --- | --- |
| Doses.rus.gp | Observed Russell et al guinea pig dose data; number of bacteria | Doses.hm | Observed Tigertt et al human dose data; egg slurry dilutions |
| N.rus.gp | Observed Russell et al number infected/ill guinea pig data | N.hm | Observed Tigertt et al number infected/ill human data |
| Doses.tig.gp | Observed Tigertt et al guinea pig dose data; egg slurry dilutions | Dose.hm | Beta Poisson estimated human doses |
| N.tig.gp | Observed Tigertt et al number infected/ill guinea pig data | K.hm | Beta Poisson estimated number of infected/ill humans |
| Dose.gp | Beta Poisson estimated guinea pig doses | Dose.dilution | Parameter relating egg slurry dilutions to number of bacteria |
| K.gp | Beta Poisson estimated number of infected/ill guinea pigs | pinf | Estimated probability of infection dependent upon estimated dose |
| a.gp | Alpha parameter of guinea pig beta Poisson model | a.gp | Alpha parameter of human beta Poisson model |
| b.gp | Beta parameter of guinea pig beta Poisson model | b.gp | Beta parameter human of beta Poisson model |

*Table SM1 Prior distributions of parameters (mean, sd) in the Bayesian framework, the model posterior.*

| Parameter | Prior | Posterior | Posterior (no data) |
| --- | --- | --- | --- |
| logeta.hm | (0, 5) | (0.10, 4.50) | (-0.01, 5.19) |
| logr.hm | (0, 5) | (2.93, 3.27) | (0.10, 4.93) |
| w.hm | (0, 5) | (-0.66, 1.80) | (0.03, 5.04) |
| z.hm | (0, 5) | (-0.54, 2.83) | (0.14, 4.95) |
| logeta.gp | (0, 5) | (1.42, 4.38) | (0.06, 5.11) |
| logr.gp | (0, 5) | (3.59, 3.29) | (0.08, 4.99) |
| w.gp | (0, 5) | (-3.16, 0.61) | (-0.06, 4.96) |
| z.gp | (0, 5) | (5.91, 2.51) | (0.03, 5.07) |
| logconc.tig | (14, 14) | (12.7, 0.70) | (14.1, 14.07) |
| logresp.tig.hm | (1.96, 0.17) | (7.15, 1.19) | (1.96, 0.17) |
| logresp.tig.gp | (-1.68, 0.31) | (0.20, 0.066) | (-1.68, 0.30) |

*Code SM1 JAGS model used representing the Bayesian framework.*

model {

# ancestor nodes: hyperparameters

# guinea pigs

# symptoms

logeta.gp ~ dnorm(mu.logeta.gp, 1/si.logeta.gp^2);

logr.gp ~ dnorm(mu.logr.gp, 1/si.logr.gp^2);

eta.gp <- exp(logeta.gp);

r.gp <- exp(logr.gp);

# infection

w.gp ~ dnorm(mu.w.gp, 1/si.w.gp^2);

z.gp ~ dnorm(mu.z.gp, 1/si.z.gp^2);

u.gp <- exp(w.gp) / (1+exp(w.gp));

v.gp <- exp(z.gp);

a.gp <- u.gp*v.gp;

b.gp <- (1-u.gp)*v.gp;

# Human volunteers

# symptoms

logeta.hm ~ dnorm(mu.logeta.hm, 1/si.logeta.hm^2);

logr.hm ~ dnorm(mu.logr.hm, 1/si.logr.hm^2);

eta.hm <- exp(logeta.hm);

r.hm <- exp(logr.hm);

# infection

w.hm ~ dnorm(mu.w.hm, 1/si.w.hm^2);

z.hm ~ dnorm(mu.z.hm, 1/si.z.hm^2);

u.hm <- exp(w.hm) / (1+exp(w.hm));

v.hm <- exp(z.hm);

a.hm <- u.hm*v.hm;

b.hm <- (1-u.hm)*v.hm;

logresp.tig.gp ~ dnorm(mu.logresp.tig.gp,1/si.logresp.tig.gp^2);

resp.tig.gp <- exp(logresp.tig.gp);

logresp.tig.hm ~ dnorm(mu.logresp.tig.hm,1/si.logresp.tig.hm^2);

resp.tig.hm <- exp(logresp.tig.hm);

logconc.tig ~ dnorm(mu.logconc.tig,1/si.logconc.tig^2);

conc.tig <- exp(logconc.tig);

# Russell guinea pig dose response

for(ind.rus.gp in 1:dosenum.rus.gp) {

# infection

dose.rus.gp[ind.rus.gp] ~ dpois(doses.rus.gp[ind.rus.gp]);

gamma.rus.gp[ind.rus.gp] <- loggam(a.gp+b.gp) -

loggam(a.gp+b.gp+dose.rus.gp[ind.rus.gp]) +

loggam(b.gp+dose.rus.gp[ind.rus.gp]) -

loggam(b.gp);

prinf.rus.gp[ind.rus.gp] <- (1-exp(gamma.rus.gp[ind.rus.gp]));

infec.rus.gp[ind.rus.gp] ~ dbin(prinf.rus.gp[ind.rus.gp],expos.rus.gp[ind.rus.gp]);

# conditional illness

prill.rus.gp[ind.rus.gp] <- (1-pow(1+eta.gp*dose.rus.gp[ind.rus.gp],-r.gp));

sympt.rus.gp[ind.rus.gp] ~ dbin(prinf.rus.gp[ind.rus.gp]*prill.rus.gp[ind.rus.gp],expos.rus.gp[ind.rus.gp]);

}

# Tigertt guinea pig dose response

for(ind.tig.gp in 1:dosenum.tig.gp) {

# infection

dose.tig.gp[ind.tig.gp] ~ dpois(conc.tig*resp.tig.gp*dilutions.tig.gp[ind.tig.gp]);

gamma.tig.gp[ind.tig.gp] <- loggam(a.gp+b.gp) -

loggam(a.gp+b.gp+dose.tig.gp[ind.tig.gp]) +

loggam(b.gp+dose.tig.gp[ind.tig.gp]) -

loggam(b.gp);

prinf.tig.gp[ind.tig.gp] <- (1-exp(gamma.tig.gp[ind.tig.gp]));

infec.tig.gp[ind.tig.gp] ~ dbin(prinf.tig.gp[ind.tig.gp],expos.tig.gp[ind.tig.gp]);

# conditional illness

prill.tig.gp[ind.tig.gp] <- (1-pow(1+eta.gp*(dose.tig.gp[ind.tig.gp]),-r.gp));

sympt.tig.gp[ind.tig.gp] ~ dbin(prinf.tig.gp[ind.tig.gp]*prill.tig.gp[ind.tig.gp],expos.tig.gp[ind.tig.gp]);

}

# Tigertt human dose response

for(ind.tig.hm in 1:dosenum.tig.hm) {

# infection

dose.tig.hm[ind.tig.hm] ~ dpois(conc.tig*resp.tig.hm*dilutions.tig.hm[ind.tig.hm]);

gamma.tig.hm[ind.tig.hm] <- loggam(a.hm+b.hm) -

loggam(a.hm+b.hm+dose.tig.hm[ind.tig.hm]) +

loggam(b.hm+dose.tig.hm[ind.tig.hm]) -

loggam(b.hm);

prinf.tig.hm[ind.tig.hm] <- (1-exp(gamma.tig.hm[ind.tig.hm]));

infec.tig.hm[ind.tig.hm] ~ dbin(prinf.tig.hm[ind.tig.hm],expos.tig.hm[ind.tig.hm]);

# conditional illness

prill.tig.hm[ind.tig.hm] <- (1-pow(1+eta.hm*(dose.tig.hm[ind.tig.hm]),-r.hm));

sympt.tig.hm[ind.tig.hm] ~ dbin(prinf.tig.hm[ind.tig.hm]*prill.tig.hm[ind.tig.hm],expos.tig.hm[ind.tig.hm]);

}

} # end model
